# Patient safety culture and associated factors: A quantitative and qualitative study of healthcare workers’ view in Jimma zone Hospitals, Southwest Ethiopia

**DOI:** 10.1186/s12913-016-1757-z

**Published:** 2016-09-20

**Authors:** Sintayehu Daba Wami, Amsalu Feleke Demssie, Molla Mesele Wassie, Ansha Nega Ahmed

**Affiliations:** 1 Department of Environmental and Occupational Health and Safety, Institute of Public Health, University of Gondar, Gondar, Ethiopia; 2 Department of Health Service Management and Health Economics, Institute of Public Health, University of Gondar, Gondar, Ethiopia; 3 Department of Human Nutrition, Institute of Public Health, University of Gondar, Gondar, Ethiopia

**Keywords:** Patient safety culture, Jimma Zone Hospitals, Ethiopia

## Abstract

**Background:**

Patient safety culture is an important aspect for quality healthcare delivery and is an issue of high concern globally. In Ethiopia health system little is known and information is limited in scope about patient safety culture. Therefore, the aim of this study was to assess the level of patient safety culture and associated factors in Jimma zone Hospitals, southwest Ethiopia.

**Methods:**

Facility based cross sectional quantitative study triangulated with qualitative approaches was employed from March to April 30/2015. Stratified sampling technique was used to select 637 study participants among 4 hospitals. The standardized tool which measures 12 patient safety culture composites was used for data collection. Bivariate and multivariate linear regression analyses were performed using SPSS version 20. Significance level was obtained at 95 % CI and *p*-value < 0.05. Semi structured guide in depth interview was used to collect the qualitative data. Content analysis of the interview was performed.

**Results:**

The overall level of patient safety culture was 46.7 % (95 % CI: 43.0, 51.2). Hours worked per week (β =−0.06, 95 % CI:−0.12,−0.001), reporting adverse event (β = 3.34, 95 % CI: 2.12, 4.57), good communication (β = 2.78, 95 % CI: 2.29, 3.28), teamwork within hospital (β = 1.91, 95 % CI: 1.37, 2.46), level of staffing (β = 1.32, 95 % CI: 0.89, 1.75), exchange of feedback about error (β = 1.37, 95 % CI: 0.91, 1.83) and participation in patient safety program (β = 1.3, 95 % CI: 0.57, 2.03) were factors significantly associated with the patient safety culture. The in depth interview indicated incident reporting, resources, healthcare worker attitude and patient involvement as important factors that influence patient safety culture.

**Conclusions:**

The overall level of patient safety culture was low. Working hours, level of staffing, teamwork, communications openness, reporting an event and exchange of feedback about error were associated with patient safety culture. Therefore, interventions of systemic approach through facilitating opportunities for communication openness, cooperation and exchange of ideas between healthcare workers are needed to improve the level of patient safety culture.

**Electronic supplementary material:**

The online version of this article (doi:10.1186/s12913-016-1757-z) contains supplementary material, which is available to authorized users.

## Background

Patient safety culture is an important aspect for quality of healthcare delivery and is an issue of high concern globally [1–5]. Patient safety culture is the product of individual and group values, attitudes, perceptions, competencies, and patterns of behavior that determine the commitment, the style and proficiency of the health providers’ safety management [2, 6, 7].

In Africa little is known and information are limited in scope about patient safety culture [8, 9]. The study in Ethiopia, which used patient safety culture survey tool of Agency of Healthcare Research and Quality, showed very low positive patient safety grade (excellent 7.2 % and very good 20.7 %) [10].

Adverse events due to unsafe practice in the process of medical care represent a major source of morbidity and mortality globally [11]. Even though evidences are limited, the possibility of patients being harmed in hospitals when receiving medical care is known to be large in African health systems [12, 13].

Establishing a culture of patient safety in the healthcare is essential to improve quality of care and promote patient safety [2, 14–17]. Many factors can be associated with patient safety culture. Organizational culture that encourages reporting and avoids blame and improved communication are reported as important factors to improve patient safety culture [18]. Other studies showed that patient safety culture can be influenced by respondent and hospital characteristics, work area, position, extent of participation in a patient safety program, communication, patient safety management and resources [19–21].

Inappropriate funding and unavailability of critical support systems including strategies, guidelines, tools and patient safety standards remain major worries in Africa. Furthermore, understanding of the problems associated with patient safety culture is hampered by inadequate data [8].

In Ethiopia health system there is also little empirical evidence about patient safety culture and associated factors.

Therefore, this study aimed to assess the level of patient safety culture and find out the associated factors in Jimma zone Hospitals, Southwest Ethiopia.

## Methods

### Study design, setting and sample

A cross sectional quantitative study triangulated with qualitative approaches was employed from March to April 30/2015.

The study was conducted among 4 hospitals, found in Jimma zone, Southwest of Ethiopia. The population being served by the hospitals is estimated to be more than five million including people from border zones and southern part of Sudan. The hospitals deliver health services in many specialty areas. These include gynecology and obstetrics, surgery, pediatrics and child health, internal medicine, ophthalmology, psychiatry, and dentistry.

Healthcare workers in Jimma zone hospitals, who had worked at least for 6 months in the hospitals, were included in this study. Stratified sampling technique was used to select the 637 study participants among 4 hospitals. Healthcare workers were stratified based on the size of hospital. The number of sample points was determined by using proportional allocation formula for each stratum. Then, the required sample sizes were selected by using simple random sampling technique from each stratum. This study targeted clinical and non-clinical staff of hospitals, like physicians, nurses, midwives, psychiatrist, pharmacy, laboratory staff, radiology staff, supervisors, and hospital managers.

For the qualitative study 10 participants were interviewed based on purposive sampling.

### Data collection procedure and tool

Quantitative data was collected through self-administered data collection technique.

The one-to-one in depth interview using a semi-structured guide was performed to collect qualitative data.

The questionnaire was originally in English version and it was translated to Amharic and back to English by another translator to check the consistence of message from question. The translation was then reviewed by professional experts. Before starting the actual data collection, 10 % of questionnaires were pre-tested in order to check the validity and consistency of the Amharic translated version of the questionnaire.

The Hospital Survey on Patient Safety Culture (HSOPSC) was used to collect quantitative data on patient safety culture. The tool was designed to assess hospital staff opinions about patient safety culture. It includes 42 items that measure 12 dimensions or composites of patient safety culture. Most items use the 5-point likert response scale of agreement (strongly disagree to strongly agree) or frequency (never to always) [22].

The tool also includes two questions in which respondents provide an overall grade on patient safety in their work area/unit and to indicate the number of events they have reported over the past 12 months (See Additional file 1).

Internal consistency/reliability was checked by calculating Cronbach’s alpha for each of the composite to ensure that items with in each composite were consistent. In this study the Cronbach’s alpha for the composites ranged from 0.61 to 0.88. The HSOPSC user’s guide indicate that a value of Cronbach’s alpha 0.60 or greater is assumed to be acceptable [22]. Therefore, each of the dimensions was found to have an acceptable reliability (See Table 4).

For the qualitative data semi structured guide in depth interview was conducted. The in depth interview was focused to identify factors influencing patient safety culture (See Additional file 2).

Six Bachelor of Science graduate nurses’ data collectors with three supervisors were assigned for quantitative data collection. The qualitative data was collected by the principal investigator.

### Measurements

**Level of patient safety culture-**measured by the healthcare workers response on the HSOPSC questionnaire in likert scale and percentages of the positive responses (strongly agree and agree or Most of the time and Always) for the 12 patient safety culture dimensions (42 items) were considered as overall level of patient safety culture.

- Scores of 75 % and above considered as good patient safety culture/area of strength.
- Scores between 50 % and 75 % considered as neutral patient safety culture.
- Scores of less than 50 % considered as poor/low patient safety culture/need improvement.

Composite level scores were computed by summation of the items within the composite scales and dividing by the number of items. Negatively worded items were reversed when computing percent positive response.

In this study event means any type of error, mistakes, incident, near misses, accident or deviation regardless of whether or not it results in patient harm.

### Data management and analysis

Quantitative data was checked, edited, coded and entered to Epi-info version 7.00 and exported to Statistical Package for Social Science (SPSS) version 20 for further analysis.

A one-way analysis of variance (ANOVA) was conducted on each of the safety culture dimensions to determine the extent to which composite scores on these safety culture scales are differentiated across hospitals. Linear regression model was fitted to identify factors associated with patient safety culture. The patient safety culture was regressed against the demographic, socio-economic and systemic factors. Before fitting linear regression model, first the assumptions were checked.

Accordingly, the assumption of linearity checked through both scatter plot and lack of fit test and it was satisfied. The assumption of normality checked by plotting histogram and P-P plots and it was also satisfied. The assumption of homoscedasticity was satisfied by plotting scatter plot of standardized residuals against the standardized predicted values and it was randomly distributed as shown in SPSS output.

The Durbin Watson statistics was used to check the assumption of independence of errors and autocorrelations. The value of the Durbin Watson statistics for this data was 2.00 which fall within the acceptable range from 1.50 to 2.50; therefore this analysis satisfied the assumption of independence and no autocorrelations. Multicollinearity assumption was checked through Variance Inflation Factor (VIF). The analysis showed VIF for each independent variable less than 10. Hence there was no evidence of Multicollinearity.

Bivariate linear regression analysis was performed and variable with *p*-value < 0.20 was exported to multivariate linear regression analysis. Significance level was obtained at 95 % CI and *p*-value < 0.05. The categorical independent variables were entered as dummy variables.

For qualitative data content analysis of the interview was performed. Tape recorded in depth interview was transcribed and then translated into English by two professionals. The translated material was read several times in order to get the general sense of the contents. Inductive approach was followed to allow the conceptual cluster of ideas and patterns to emerge. Then compared and reorganized into tentative categories. For the reporting of qualitative research we adhered to the COREQ guidelines.

## Results

Of the total 637 questionnaires distributed to different departments in the 4 hospitals 596 completed and valid questionnaires were returned, which gives a response rate of 93.6 %.

### Demographic and socio-economic characteristics of the study participants

From the total respondents, 367 (61.6 %) were males. The mean age with standard deviation of the respondents was 28.8 ± 6 years and the age of the study participants ranged from 21 to 57 years. Among 596 respondents, 301 (50.5 %) were nurses and 502 (84.2 %) respondents had less than or equal to 5 years of work experiences (See Table 1). Among the study participants, 92 (15.4 %) of them works in internal medicine department followed by surgical ward 91 (15.3 %) and 87 (14.6 %) of respondents works in many departments.

**Table 1:** Demographic and socio-economic characteristics of the study participants in Jimma zone hospitals, Southwest Ethiopia, 2015 (*N* = 596)

Variables	Frequency (n)	Percent (%)
Sex		
Male	367	61.6
Female	229	38.4
Age (years)		
≤ 29	407	68
30 - 44	170	29
≥ 45	19	3
Marital status		
Single	291	48.8
Married	255	42.8
Divorced/Widowed	50	8.4
Religion		
Orthodox	253	42.4
Muslim	169	28.4
Protestant	162	27.2
Others^a^	12	2
Work experience in current hospital (years)		
≤ 5	502	84.2
6–10	58	9.7
≥ 11	36	5.9
Profession		
Nurse	301	50.5
Medical doctor	88	14.8
Dentist	21	3.5
Pharmacist	33	5.5
Midwives	55	9.2
Psychiatrist	21	3.5
Lab technologist	35	5.9
Others^b^	42	7.1

Others ^a^: Catholic, Adventist, Waqefeta; others ^b^ = Anesthetist, Optometry, Management

Of the total 596 study participants, 514 (86.2 %) respondents had direct interaction or contact with the patients and 448 (75.2 %) of the respondents did not received any training on patient safety (See Table 2).

**Table 2:** Systemic factors and personal characteristics of the study participants in Jimma zone hospitals, Southwest Ethiopia, 2015 (*N* = 596)

Variables	Frequency (n)	Percent (%)
Direct contact with the patients		
No	82	13.8
Yes	514	86.2
Patient safety training		
No	448	75.2
Yes	148	24.8
Participation in patient safety program		
Never	298	50
At least once per year	298	50
Hospital management encourage reporting of events		
No	392	65.8
Yes	204	34.2
Hospital management blame when medical errors happened		
No	255	42.8
Yes	341	57.2
Hours worked per week (Hours)		
39–59	505	84.8
≥ 60	91	15.2
We work together as a team		
Strongly disagree/Disagree	54	9.1
Neutral	25	4.2
Agree/Strongly agree	517	86.7
There is adequate staffing		
Strongly disagree/Disagree	315	52.8
Neutral	79	13.3
Agree/Strongly agree	202	33.9
We communicate freely		
Never/Rarely	297	49.8
Sometimes	125	21
Most of the time/Always	174	29.2
Exchange of feedbacks with each other		
Never/Rarely	168	28.2
Sometimes	151	25.3
Most of the time/Always	277	46.5

### Patient safety grade and Number of events reporting

According to this study, 11 % and 23 % of the respondents rated the patient safety grade as excellent and very good, respectively. Among participants, 39 % of respondents rated patient safety grade as acceptable, while 21 % and 6 % of respondents rated the patient safety grade as poor and falling respectively.

Regarding number of events reported in the past 12 months the majority (69 %) of respondents never reported any event/error (See Additional file 3: Figure S1).

### Comparative results on patient safety culture dimensions across Jimma zone hospitals

On 7 out of 12 patient safety culture dimensions, the 4 hospitals show significant differences in their score (*P*-Value < 0.05). These are teamwork within hospital departments, supervisor/manager expectations and actions promoting safety, organizational learning/continuous improvement, hospital management support for patient safety, frequency of event reporting, teamwork across hospital departments and hospital handoffs and transitions. This variation might be due to the differences in location and size of hospitals, level of staffing, style of leadership, management strategy and relationships with in hospital staff between hospitals (See Table 3).

**Table 3:** Comparative results on Patient safety culture dimensions across Jimma zone hospitals, Southwest Ethiopia, 2015

Patient safety culture dimensions	average positive responses				*P*-Value
	JUSH (*N* = 442)	SGH (*N* = 54)	LGH (*N* = 58)	AH (*N* = 42)	
Team work with in hospital	82 %	89 %	79 %	80 %	0.038
Supervisor expectation	47 %	60 %	48 %	45 %	0.024
Organizational learning	74 %	75 %	55 %	60 %	<0.001
Mgt support for patient safety	41 %	49 %	50 %	40 %	0.021
Perception of patient safety	50 %	58 %	47 %	52 %	0.199
Feedback & communication about error	35 %	31 %	22 %	29 %	0.095
Communication openness	47 %	49 %	34 %	46 %	0.36
Frequency of event reporting	28 %	31 %	21 %	20 %	<0.001
Teamwork across hospital	57 %	69 %	65 %	66 %	0.012
Staffing	35 %	40 %	31 %	33 %	0.552
Handoffs and transitions	43 %	50 %	34 %	29 %	0.032
Non punitive response to error	23 %	28 %	30 %	17 %	0.163

*N* sample size for each hospital, *JUSH* Jimma University Specialized Hospital, *LGH* Limu Genet Hospital, *AH* Agaro Hospital

### Overall level of patient safety culture

The overall level of patient safety culture was 46.7 % (95 % CI: 43.0, 51.2). The dimension with the highest average percentage positive responses was teamwork within department (82 %). While the area with the most potential for improvement and the lowest average percentage positive responses was non-punitive response to error (23.7 %) (See Table 4).

**Table 4:** Patient safety culture composite level results of Jimma zone hospitals, Southwest Ethiopia, 2015 (*N* = 596)

Patient safety culture dimensions	Number of Items	Cronbach’s alpha	Positive safety culture score
Teamwork within hospital departments	4	0.802	82 %(74–89)
Organizational learning	3	0.79	71.3 %(61–87)
Teamwork across hospital departments	4	0.72	59.5 %(49–70)
Overall perception of patient safety	4	0.62	50.5 %(36–62)
Supervisor expectation and action promoting safety	4	0.63	48.5 %(38–55)
Communication openness	3	0.65	46 %(45–47)
Hospital management support for patient safety	3	0.68	42.7 %(41–44)
Hospital handoffs and transitions	4	0.81	41.5 %(35–47)
Staffing	4	0.608	35.25 %(27–44)
Feedback and communication about error	3	0.80	33 %(28–37)
Frequency of event reporting	3	0.88	27 %(25–30)
Non punitive response to error	3	0.74	23.7 %(20–29)
Overall level of patient safety culture	42	0.84	46.7 %

### Factors associated with patient safety culture

The multivariable analysis model explained 59 % of the variance in the patient safety culture (Adjusted R square = 0.59, *P*-Value < 0.001).

Multivariate analysis showed that hours worked per week, level of staffing, teamwork within hospital, good communication, reporting an event, exchange of feedback about error and participation in patient safety program were found to be significantly associated with the patient safety culture (See Table 5).

**Table 5:** Factors associated with patient safety culture in Jimma zone hospitals, Southwest Ethiopia, 2015 (*N* = 596) (Multivariate Linear Regression analysis)

Variables	No. (%)	Unstand. Coeff. (β)	SE	95 % CI for β
Sex				
Female^1^	229 (38.4)	0		
Male	367 (61.6)	−0.71	0.75	(−0.943, 0.346)
Age (years)				
≤ 29^1^	407 (68)	0		
30–44	170 (29)	0.71	0.69	(−.644, 2.058)
≥ 45	19 (3)	−.06	2.02	(−4.032, 3.912)
Direct contact with patients				
No^1^	82 (14)	0		
Yes	514 (86)	−0.82	0.96	(−2.701, 1.071)
Primary work department				
Medical^1^	92 (15.4)	0		
Many different department	87 (14.6)	0.45	0.99	(−1.507, 2.415)
Surgical	91 (15.3)	1.75	0.97	(−0.162, 3.654)
Obstetrics	66 (11.1)	3.32	1.80	(−0.216, 6.861)
Pediatrics	48 (8.1)	0.29	1.14	(−1.939, 2.534)
Outpatient department	41 (6.9)	−1.59	1.21	(−3.964, 0.788)
Psychiatry/Mental health	32 (5.4)	0.26	2.04	(−3.749, 4.264)
Intensive care unit	27 (4.5)	1.63	1.47	(−1.248, 4.509)
Hours worked per week	596 (100)	−0.06	0.03	(−0.123, −0.001)*
Patient safety training				
No^1^	448 (75.2)	0		
Yes	148 (24.8)	−0.59	0.66	(−1.891, 0.715)
Years of experience at hospital				
≤ 5^1^	502 (84.2)	0		
6–10	58 (9.7)	−1.44	1.01	(−3.428, 0.555)
≥ 11	36 (6.1)	−1.47	1.54	(−4.490, 1.554)
Adverse event report				
No^1^	370 (62.1)	0		
Yes	226 (37.9)	3.34	0.62	(2.119, 4.567)**
Teamwork within hospital	596 (100)	1.91	0.28	(1.366, 2.457)**
Communication with each other	596 (100)	2.78	0.25	(2.285, 3.284)**
Level of staffing	596 (100)	1.32	0.22	(0.885, 1.746)**
Feedback when error happened	596 (100)	1.37	0.23	(0.914, 1.831)**
Management encouragement				
No^1^	392 (65.8)	0		
Yes	204 (34.2)	0.88	0.64	(−0.372, 2.125)
Participation in patient safety program				
No^1^	298 (50)	0		
Yes	298 (50)	1.30	0.37	(0.573, 2.031)**

^1^ = Reference group, *P-Value <0.05, **P-Value < 0.001, *No*. Number

### Finding from the qualitative study

A total of 10 health care professionals were successfully interviewed. Of who interviewed 4 were nurses and others were from physicians, midwives and clinical pharmacy profession represented by 2 respondents from each.

The factors influencing patient safety culture as perceived by health care professionals were thematically categorized as system factors, health care professional factors and patient factors.

### System factors

Respondents believed that event reporting system positively affect patient safety culture. Event reporting was described as one type of safety information system that relies on staff reports of errors, safety concerns, adverse events and near misses that occur within routine situations to learn from it. Event reporting was mentioned as an essential component by health care workers to improve learning culture and then patient safety culture. However, they highlighted that event reporting system was not established in the hospital and most of professionals in the hospital never reported the event.

“I remember one case when I had been working in surgical department, one patient dead due to health care professional mistakes but they kept silent about the case, you see it was forgotten now without learning from that mistakes and not sure the same mistakes will not occur in the future” (R09, Nurse)

Participants described shortage of supply and equipment as the factors which hinder patient safety culture. They highlighted shortage of water supply, lack of gloves, syringes, emergency drugs and cabinet shelves which seems simple but affecting the patient safety culture strongly.

“Sometimes gloves and syringes which seems simple were not found when we try to give medication” (R02, physician)

“In maternity department water supply is very necessary but there is shortage” (R01, Midwives)

Majority of respondents expressed as shortage of staff and high workload on health care professionals negatively affect the patient safety culture. They believed infrastructures of hospital and people served should be balanced to provide safe care.

“…For example, there is no personnel responsible for patient safety in this hospital, in developed countries I know there is risk management department, rather here limited to infection prevention”(R02, physician)

“I am amazed that one physician should see up to 30 patients per day, how you can decide the length of duration for one case, this shows shortage of staff” (R03, physician)

### Health care professional’s factors

Respondents believed conflict between professionals and poor teamwork negatively affect patient safety culture. Collaboration of health professionals and respecting each other is an important factor. Some nurses reported lack of collaboration between physicians and non-physicians looked as a barrier to improve patient safety culture due to hierarchical differences.

“In my opinion patient safety is improved by teamwork/collaboration between health care professionals, but in this hospital I am afraid to say there is teamwork and respect between physicians and non-physicians professionals.” (R04, Nurse)

The low attitude of healthcare professionals toward patient safety was reported to affect patient safety culture negatively. Respondents highlighted the perception of healthcare professionals on patient safety as very low.

*“…When I say low attitude of health care professionals, I mean internal feeling/interest of professionals on* patient safety culture *is very low, this may affect the patient safety culture negatively” (R06, Nurse)*

“I think health care professional’s attitude toward patient safety is poor, this may be due to lack of training.” (R03, Physician)

### Patient factors

Patient perception about the health service provided and their interaction also influence the patient safety culture according to the respondents thought. Most of the respondents were reported as patient involvement is very important for achieving positive patient safety culture.

“Majority of patients do not know who to contact to get the service they need and do not interact while they feel unsafe in a situation, this might be due to lack of awareness of patient safety” (R08, Clinical pharmacist)

In general all respondents believed government, hospital management, each hospital staff and patient itself are responsible to achieve increased patient safety culture.

Increased awareness of patients on service, positive attitude of health care professionals on patient safety, improved communication between healthcare professionals, increased teamwork and adequate supply chain were important factors to achieve better patient safety culture as described by respondents.

## Discussion

In this study the overall level of patient safety culture was found to be 46.7 % (95 % CI: 43.0, 51.2). This result showed that the hospitals had poor/low patient safety culture and areas with the most potential for improvement and needs urgent improvements. This result is comparable with the study reported 48 % in India [23]. This similarity might be due to the similarities in staffing and hospital infrastructure between countries.

However, the overall level of patient safety culture of this study is lower when compared with the study reported, 51.75 % in Japan [24], 52.2 % in Netherlands [25], 52.9 % in Taiwan [24], 52.8 % in Iran [26], 62 % in USA [25], 62.7 % in Srilanka [27], 64 % in another study conducted in Taiwan [14] and 65 % in China [28]. This difference might be due to the differences in organizational behavior between countries. Those countries might have better management values, organizational commitments, leadership and relationships within hospital staff. Other possible reasons might be due to high economic development and those countries were initiated patient safety issue early compared to our country.

In this study ‘teamwork within department’ and ‘organizational learning’ dimensions were area of strength with average positive response rate of 82 % and 71.3 % respectively. This indicates respondents are positive in supporting one another, working together as a team and doing things to improve patient safety.

Although respondents generally described as there is good team work within departments, poor communication and collaboration between professions within hospitals was described by few respondents during the in depth interview. This weak communication was explained due to hierarchical differences. This result is in line with the finding of a qualitative study in two African hospitals [29]. This result indicates patient safety culture is influenced by the norms and values of professional thought and efforts to regain status.

The areas with the most potential for improvement in this study area were ‘non punitive response to error’ and ‘frequency of event reporting’ with average positive response rate of 23.7 % and 27 % respectively. This result suggest healthcare workers in this study area feel that their mistakes and event reports are held against them and that mistakes are kept in their personal file.

Dimensions like ‘feedback and communication about error’, ‘Staffing’, ‘Hospital handoffs and transitions’, ‘hospital management support for patient safety’, ‘communication openness’ and ‘supervisor/manager expectation and actions promoting safety’ were areas with potential for improvement with average positive response rate of 33 %, 35.25 %, 41.5 %, 42.7 %, 46 % and 48.5 % respectively. This finding highlights deficiencies in many patient safety culture dimensions and indicates presence of low patient safety practice in the study area.

For every unit increase in hours worked per week the patient safety culture score found to reduce by 0.06 (β =−0.06, 95 % CI:−0.12,−0.001). This result strengthened by qualitative findings where respondents believed when healthcare professionals work longer hours they try to work faster than best for patient care. This may be explained as when hospital staff worked longer hours, the way of working by trying to do too quickly may have negative impact on performance, and consequently has poor patient safety culture.

This study showed, respondents who report an event/error either orally or in written form increase the score of patient safety culture by 3.34 compared to respondents not reporting an event (β = 3.34, 95 % CI: 2.12, 4.57). This result is also supported by the qualitative finding, in which respondents believed reporting an event has a positive impact on patient safety culture. This finding is in line with the result reported in Lebanon [19] and Sweden [18]. This might be due to the truth that event reporting systems generate useful information to address weakness in work systems and processes, so it provides an opportunity to improve the patient safety culture.

Participating in patient safety program increased the patient safety culture score by 1.3 (β = 1.3, 95 % CI: 0.57, 2.03). This result is in line with the study reported in Sweden [30]. This result might be explained as the staff used more times on patient safety issues in patient safety program, this situation favor them to deal more with patient safety culture.

This study found that a one unit increase in the score on teamwork within hospital improved the patient safety culture score by 1.91 (β = 1.91, 95 % CI: 1.37, 2.46). The findings from qualitative study also support these results, respondents felt increased teamwork and collaboration between health care professionals improves patient safety culture. This finding is similar with studies conducted in Lebanon [19] and Riyadh [31], which reported as teamwork within department increase perception of patient safety culture. This might be explained as higher score on people support one another, working together as a team and respecting each other improve the patient safety culture.

According to this study, for every unit increase on the score of communication with each other the patient safety culture score improved by 2.78 (β = 2.78, 95 % CI: 2.29, 3.28). This finding is again in line with the study in Lebanon [19], which state higher score on communication openness increase perception of patient safety culture. But the study in Riyadh [31] had showed for a one unit increase in communication openness was associated with lower perception of patient safety culture. This variation might be due to the difference in the national culture of the countries.

As per this study, overall patient safety culture score improved by 1.32 for every unit increase in the score on level of staffing (β = 1.32, 95 % CI: 0.89, 1.75). The result is analogous to study conducted in Lebanon [19] and Riyadh [31], where higher score on staffing associated with improved perception of patient safety culture. This finding may indicate as staffs within departments adequate to handle the workload the patient safety culture could be improved.

Similarly the finding from the qualitative approach showed shortage of staff in the hospitals negatively affects the patient safety culture. As the staff works additional or extra-long times the probability of practicing better patient safety culture is low. This finding is in line with result of the qualitative study conducted in two African hospitals [29]. This result suggests hospitals in African regions are characterized by shortage of staff.

Based on this study for every unit increase in exchange of feedback when error happened increases the score on patient safety culture by 1.37 (β = 1.37, 95 % CI: 0.91, 1.83). This finding is supported by the qualitative results; where respondents believed investigation of the root cause of error and exchange of comprehensive feedback of error improve the patient safety culture.

From the qualitative finding respondents believed patient involvement and health care professional’s attitude toward patient safety influence patient safety culture. This result is supported by qualitative study conducted in Sweden [30].

Since we assessed patient safety culture from hospitals staff opinion, the possibility of under or over reporting could not be ruled out. But through honestly explaining the objective and significances of the study we tried to minimize the effect.

## Conclusions

The finding of this study showed that the overall level of patient safety culture in Jimma zone hospitals found to be low.

Hours worked per week, adverse event reporting, good communication, teamwork within hospital, level of staffing, exchange of feedback about error and participating in patient safety program were factors significantly associated with the patient safety culture. Hours worked per week, incident reporting, teamwork, level of staffing and exchange of feedback about error were supported by the qualitative finding.

While shortage of resource, healthcare professional attitude toward patient safety and patient involvement were additional variables explored as important factors that influence patient safety culture during in depth interview.

## Additional files

Additional file 1: Questionnaire to assess Patient Safety Culture and Associated Factors. The HSOPSC was used to collect quantitative data on patient safety culture. The questionnaire was classified into 3 parts and * part questions were added when adapting HSOPSC. (DOCX 53 kb) Additional file 2: Semi structured interview guide questions. The in depth interview was focused to identify factors influencing patient safety culture. (DOCX 14 kb) Additional file 3: Figure S1. Number of events reported by respondents in Jimma zone hospitals, Southwest Ethiopia, 2015 (*N* = 596). (DOCX 14 kb)

## Abbreviations

HSOPSC: Hospital survey on patient safety culture
SPSS: Statistical package for social science
USA: United State of America
VIF: Variance inflation factor
