# Supplementary material for: Patient safety culture and associated factors: A quantitative and qualitative study of healthcare workers’ view in Jimma zone Hospitals, Southwest Ethiopia
Source: BMC Health Serv Res. 2016 Sep 20;16:495. doi: 10.1186/s12913-016-1757-z (PMC5029028; doi:10.1186/s12913-016-1757-z)
Supplement: Additional file 1: — Questionnaire to assess Patient Safety Culture and Associated Factors. The HSOPSC was used to collect quantitative data on patient safety culture. The questionnaire was classified into 3 parts and * part questions were added when adapting HSOPSC. (DOCX 53 kb) [file 12913_2016_1757_MOESM1_ESM.docx]

**Questionnaire for Patient Safety Culture and Associated Factors: A Quantitative and Qualitative Study of Healthcare Workers' View in Jimma Zone Hospitals, Southwest Ethiopia, 2015**

Hospital Name _______________________ Bed Numbers_____________

Working department_____________________________

**Part I: - SOCIO-DEMOGRAPHIC**

| **S.NO** | **Questions** | **Response** | **Code** |
| --- | --- | --- | --- |
| 101 | ID |  |  |
| 102 | Sex | Female---------------0  Male-------------------1 |  |
| 103 | Age in Year |  |  |
| 104 | Marital Status | Single----------------0  Married---------------1  Divorced-------------2  Widowed------------3  Separated-----------4 |  |
| 105 | Religion | Orthodox-----------------0  Muslim--------------------1  Protestant----------------2  Others______________ |  |
| 106 | Educational status | Below and High school --------0  Diploma-----------------------------1  Bachelor Degree-----------------2  Master Degree--------------------3  Medical doctor--------------------4  Specialization----------------------5  Doctorate (PhD)-------------------6  Other______________________ |  |
| 107 | Profession | Medical doctor-------------------0  Dentist------------------------------1  Nurse-----------------------------2  Pharmacist-----------------------3  Midwive---------------------------4  Radiology -------------------------5  Lab technician ------------------6  Anesthetist-----------------------7  Psychiatry------------------------8  Physiotherapy ------------------9  Health officer--------------------10  Env’tal/Occupational health----11  Others__________________ |  |
| 108 | What is your primary work unit/ department or clinical area of the hospitals where you spent most of the work time or provides most of the clinical service? | Many different work area-------0  Medicine/non surgical-----------1  Surgery------------------------------2  Obstetrics--------------------------3  Pediatrics---------------------------4  Emergency dept.------------------5  Psychiatry/mental health-------6  Rehabilitation----------------------7  Pharmacy--------------------------8  Laboratory-------------------------9  Radiology--------------------------10  Anesthesiology-------------------11  Other______________________ |  |
| 109 | Years of experience in this hospital | _______________ |  |
| 110 | How long have you worked in your current hospital work area? | ______________ |  |
| 111 | In average how many hours per week do you work in this hospital? | _____________ |  |
| 112 | In your staff position do you typically have direct interaction or contact with patients? | No----------------0  Yes --------------1 |  |

**Part II: Patient safety culture dimensions**

**Section A: Your work area/unit** (Please answer the following about your work area)

| **Team work within Hospital Units** | | | | | | | | | | | | | | | | |
| --- | --- | --- | --- | --- | --- | --- | --- | --- | --- | --- | --- | --- | --- | --- | --- | --- |
| **S.NO** | **Questions** | **Response** | | | | | | | | | | | | | | **Code** |
|  |  | **Strongly**  **Disagree (SD)** | | **Disagree (D)** | | | | **Neutral(N)** | | | **Agree(A)** | | | **Strongly Agree(SA)** | |  |
| 201 | People support one another in this unit | 1 | | 2 | | | | 3 | | | 4 | | | 5 | |  |
| 202 | When a lot of work needs to be done quickly, we work together as a team to get the work done | 1 | | 2 | | | | 3 | | | 4 | | | 5 | |  |
| 203 | In this unit, people treat each other with respect. | 1 | | 2 | | | | 3 | | | 4 | | | 5 | |  |
| 204 | When one area in this unit gets really busy, others help out | 1 | | 2 | | | | 3 | | | 4 | | | 5 | |  |
| **Organizational learning – Continuous improvement** | | | | | | | | | | | | | | | | |
| **S.NO** | **Questions** | **Response** | | | | | | | | | | | | | **Code** | |
|  |  | **SD** | **D** | | | | **N** | | | **A** | | | **SA** | |  |  |
| 205 | We are actively doing things to improve patient safety | 1 | 2 | | | 3 | | | 4 | | | 5 | | |  | |
| 206 | Mistakes have led to positive changes here | 1 | 2 | | | 3 | | | 4 | | | 5 | | |  | |
| 207 | After we make changes to improve patient safety, we evaluate their effectiveness | 1 | 2 | | | 3 | | | 4 | | | 5 | | |  | |
| **Non Punitive response to error** | | | | | | | | | | | | | | | | |
| **S.NO** | **Questions** | **Response** | | | | | | | | | | | | | **Code** | |
|  |  | **SD** | **D** | | **N** | | | | | **A** | | | **SA** | |  |  |
| 208 | Staff feel like their mistakes are held against them | 1 | 2 | | 3 | | | | | 4 | | | 5 | |  | |
| 209 | When an event is reported, it feels like the person is being written up, not the problem | 1 | 2 | | 3 | | | | | 4 | | | 5 | |  | |
| 210 | Staff worry that mistakes they make are kept in their personnel file | 1 | 2 | | 3 | | | | | 4 | | | 5 | |  | |
| **Staffing** | | | | | | | | | | | | | | | | |
| **S.NO** | **Questions** | **Response** | | | | | | | | | | | | | **Code** | |
|  |  | **SD** | **D** | | | | **N** | | | **A** | | | **SA** | |  |  |
| 211 | We have enough staff to handle the workload | 1 | 2 | | | | 3 | | | 4 | | | 5 | |  | |
| 212 | Staff in this unit work longer hours than is best for patient care | 1 | 2 | | | | 3 | | | 4 | | | 5 | |  | |
| 213 | We use more agency/temporary staff than is best for patient care | 1 | 2 | | | | 3 | | | 4 | | | 5 | |  | |
| 214 | We work in “crisis mode,” trying to do too much, too quickly | 1 | 2 | | | | 3 | | | 4 | | | 5 | |  | |
| **Overall Perception of safety** | | | | | | | | | | | | | | | | |
| **S.NO** | **Questions** | **Response** | | | | | | | | | | | | | **Code** | |
|  |  | **SD** | **D** | | | | **N** | | | **A** | | | **SA** | |  |  |
| 215 | Patient safety is never sacrificed to get more work done | 1 | 2 | | | | 3 | | | 4 | | | 5 | |  | |
| 216 | Our procedures and systems are good at preventing errors from happening | 1 | 2 | | | | 3 | | | 4 | | | 5 | |  | |
| 217 | It is just by chance that more serious mistakes don’t happen around here | 1 | 2 | | | | 3 | | | 4 | | | 5 | |  | |
| 218 | We have patient safety problems in this unit | 1 | 2 | | | | 3 | | | 4 | | | 5 | |  | |

**Section B: Your supervisor/Manager (**Please answer the following about your immediate supervisor/Manager or person to whom you directly report**)**

| **Supervisor/ Manager expectations and actions promoting safety** | | | | | | | |
| --- | --- | --- | --- | --- | --- | --- | --- |
| **S.NO** | **Questions** | **Response** | | | | | **Code** |
|  |  | **SD** | **D** | **N** | **A** | **SA** |  |
| 219 | My supervisor/manager says a good word when he/she sees a job done according to established patient safety procedures | 1 | 2 | 3 | 4 | 5 |  |
| 220 | My supervisor/manager seriously considers staff suggestions for improving patient safety | 1 | 2 | 3 | 4 | 5 |  |
| 221 | Whenever pressure builds up, my supervisor/manager wants us to work faster, even if it means taking shortcuts | 1 | 2 | 3 | 4 | 5 |  |
| 222 | My supervisor/manager overlooks patient safety problems that happen over and over | 1 | 2 | 3 | 4 | 5 |  |

**Section C: Communication (**How often do the following happen in your work area?**)**

| **Communication openness** | | | | | | | | | | |
| --- | --- | --- | --- | --- | --- | --- | --- | --- | --- | --- |
| **S.NO** | **Questions** | **Response** | | | | | | | | **Code** |
|  |  | **Never (N)** | **Rarely** | | **Some times** | | **Most of the time** | | **Always** |  |
| 223 | Staff will freely speak up if they see something that may negatively affect  patient care | 1 | 2 | | 3 | | 4 | | 5 |  |
| 224 | Staff feel free to question the decisions or actions of those with more authority | 1 | 2 | | 3 | | 4 | | 5 |  |
| 225 | Staff are afraid to ask questions when something does not seem right | 1 | 2 | | 3 | | 4 | | 5 |  |
| **Feedback and communication about error** | | | | | | | | | | |
| **S.NO** | **Questions** | **Response** | | | | | | | | **Code** |
|  |  | **Never (N)** | | **Rarely** | | **Some times** | **Most of the time** | **Always** | |  |
| 226 | We are given feedback about changes put into place based on event reports | 1 | | 2 | | 3 | 4 | 5 | |  |
| 227 | We are informed about errors that happen in this unit | 1 | | 2 | | 3 | 4 | 5 | |  |
| 228 | In this unit, we discuss ways to prevent errors from happening again | 1 | | 2 | | 3 | 4 | 5 | |  |

**Section D: Frequency of Events reported (**When mistakes happen in your hospital work area, how often are they reported**)**

**Event:** Any type of error, mistakes, incident, near misses, accident or deviation regardless of whether or not it results in patient harm

| **Frequency of Event reporting** | | | | | | | |
| --- | --- | --- | --- | --- | --- | --- | --- |
| **S.NO** | **Questions** | **Response** | | | | | **Code** |
|  |  | **Never (N)** | **Rarely** | **Some times** | **Most of the time** | **Always** |  |
| 229 | When a mistake is made, but is caught and corrected before affecting the patient, how often is this reported? | 1 | 2 | 3 | 4 | 5 |  |
| 230 | When a mistake is made, but has no potential to harm the patient, how often is this reported? | 1 | 2 | 3 | 4 | 5 |  |
| 231 | When a mistake is made that could harm the patient, but does not, how often is this reported? | 1 | 2 | 3 | 4 | 5 |  |
| **Section E: Patient safety grade** | | | | | | | |
| **S.NO** | **Questions** | **Response** | | | | | **Code** |
| 232 | Please give your work area/unit in this hospital an overall grade on patient safety | Excellent--------------------1  Very good-------------------2  Acceptable------------------3  Poor---------------------------4  Falling-------------------------5 | | | | |  |

**Section F: Hospital-Wide (**please answer the following about your hospital**)**

| **Hospital Management support for patient safety** | | | | | | | | | | | | | | | |
| --- | --- | --- | --- | --- | --- | --- | --- | --- | --- | --- | --- | --- | --- | --- | --- |
| **S.NO** | **Questions** | **Response** | | | | | | | | | | | | | **Code** |
|  |  | **SD** | **D** | | | **N** | | | | **A** | | | **SA** | |  |
| 233 | Hospital management provides a work climate that promotes patient safety | 1 | 2 | | | 3 | | | | 4 | | | 5 | |  |
| 234 | The actions of hospital management show that patient safety is a top priority | 1 | 2 | | | 3 | | | | 4 | | | 5 | |  |
| 235 | Hospital management seems interested in patient safety only after an adverse event happens | 1 | 2 | | | 3 | | | | 4 | | | 5 | |  |
| **Team work across hospital unit** | | | | | | | | | | | | | | | |
| **S.NO** | **Questions** | **Response** | | | | | | | | | | | | | **Code** |
|  |  | **SD** | **D** | | | | **N** | | **A** | | | **SA** | | |  |
| 236 | There is good cooperation among hospital units that need to work together | 1 | 2 | | | | 3 | | 4 | | | 5 | | |  |
| 237 | Hospital units work well together to provide the best care for patients | 1 | 2 | | | | 3 | | 4 | | | 5 | | |  |
| 238 | Hospital units do not coordinate well with each other | 1 | 2 | | 3 | | | | 4 | | | | 5 | |  |
| 239 | It is often unpleasant to work with staff from other hospital units | 1 | 2 | | 3 | | | | 4 | | | | 5 | |  |
| **Hospital Handoffs and Transitions** | | | | | | | | | | | | | | | |
| **S.NO** | **Questions** | **Response** | | | | | | | | | | | | | **Code** |
|  |  | **SD** | | **D** | | | | **N** | | | **A** | | | **SA** |  |
| 240 | Things “fall between the cracks” when transferring patients from one unit to another | 1 | | 2 | | | | 3 | | | 4 | | | 5 |  |
| 241 | Important patient care information is often lost during shift changes | 1 | | 2 | | | | 3 | | | 4 | | | 5 |  |
| 242 | Problems often occur in the exchange of information across hospital units | 1 | | 2 | | | | 3 | | | 4 | | | 5 |  |
| 243 | Shift changes are problematic for patients in this hospital | 1 | | 2 | | | | 3 | | | 4 | | | 5 |  |
| **Section G: Number of events Reported** | | | | | | | | | | | | | | | |
| **S.NO** | **Questions** | **Response** | | | | | | | | | | | | | **Code** |
| 244 | In the past 12 months, how many event reports have you filled out and submitted? | __________________ | | | | | | | | | | | | |  |

**Part III: Factors associated with patient safety culture***

| **Workplace characteristics** | | | | | | | | |
| --- | --- | --- | --- | --- | --- | --- | --- | --- |
| **S.NO** | | **Questions** | **Response** | | | | | **Code** |
|  |  |  | **Strongly Disagre** | **Disagree** | **Neutral** | **Agree** | **Strongly Agree** |  |
| 301 | | There is good cooperation and teamwork while we give patient care | 1 | 2 | 3 | 4 | 5 |  |
| 302 | | We have adequate staff that is best for patient safety | 1 | 2 | 3 | 4 | 5 |  |
| 303 | | There is good communication with each other | 1 | 2 | 3 | 4 | 5 |  |
| 304 | | We always give feedback for each other when we saw mistakes happened | 1 | 2 | 3 | 4 | 5 |  |
| **S.NO** | **Questions** | | **Response** | | | | | **Code** |
| 305 | | Had you ever participated in patient safety program? | Never----------------------------0  Several times a year--------1  At least monthly---------------2 | | | | |  |
| 306 | | Extent of participation in patient safety issue | Never----------------------------0  Several times a year--------1  At least monthly---------------2 | | | | |  |
| 307 | | Have you ever reported adverse events? | No--------------------0  Yes-------------------1 | | | | |  |
| 308 | | Do you believe work load in this work unit increase risk for error? | No--------------------0  Yes-------------------1 | | | | |  |
| 309 | | Did you taken any patient safety training? | No--------------------0  Yes-------------------1 | | | | |  |
| 310 | | Do you think inadequacy of logistic and other resource in this hospital affect patient safety culture? | No--------------------0  Yes-------------------1 | | | | |  |
| 311 | | Does hospital management encourage reporting events? | No--------------------0  Yes-------------------1 | | | | |  |
| 312 | | Doe the hospital management blame when medical errors happened? | No-------------------0  Yes-------------------1 | | | | |  |

**Reference**

1. Sorra J, Nieva V: **Hospital survey on patient safety culture.(Prepared by Westat, under contract no. 290-96-0004). AHRQ publication no. 04-0041**. *Rockville, MD: Agency for Healthcare Research and Quality* 2004.
