# Supplementary material for: Patient safety culture and associated factors: A quantitative and qualitative study of healthcare workers’ view in Jimma zone Hospitals, Southwest Ethiopia
Source: BMC Health Serv Res. 2016 Sep 20;16:495. doi: 10.1186/s12913-016-1757-z (PMC5029028; doi:10.1186/s12913-016-1757-z)
Supplement: Additional file 2: — Semi structured interview guide questions. The in depth interview was focused to identify factors influencing patient safety culture. (DOCX 14 kb) [file 12913_2016_1757_MOESM2_ESM.docx]

**Semi structured interview questions for qualitative data**

**(**Followed by probe questions**)**

1. In your opinion what are the factors that influence/affect the patient safety culture in health care recently?

Probing question:

- How do you think these factors influence/affect the patient safety culture?
- Who do you think are responsible for these factors?

1. Based on the current level of patient safety culture in your hospital what do you think is important factor to achieve increased patient safety culture?

Followed by:

- Do you think these can be achieved at all hospital level?
- How do you think these factors incorporated with the hospitals activities?
