# Supplementary material for: Patient safety culture and associated factors: A quantitative and qualitative study of healthcare workers’ view in Jimma zone Hospitals, Southwest Ethiopia
Source: BMC Health Serv Res. 2016 Sep 20;16:495. doi: 10.1186/s12913-016-1757-z (PMC5029028; doi:10.1186/s12913-016-1757-z)
Supplement: Supplementary file 3 [file 12913_2016_1757_MOESM3_ESM.docx]

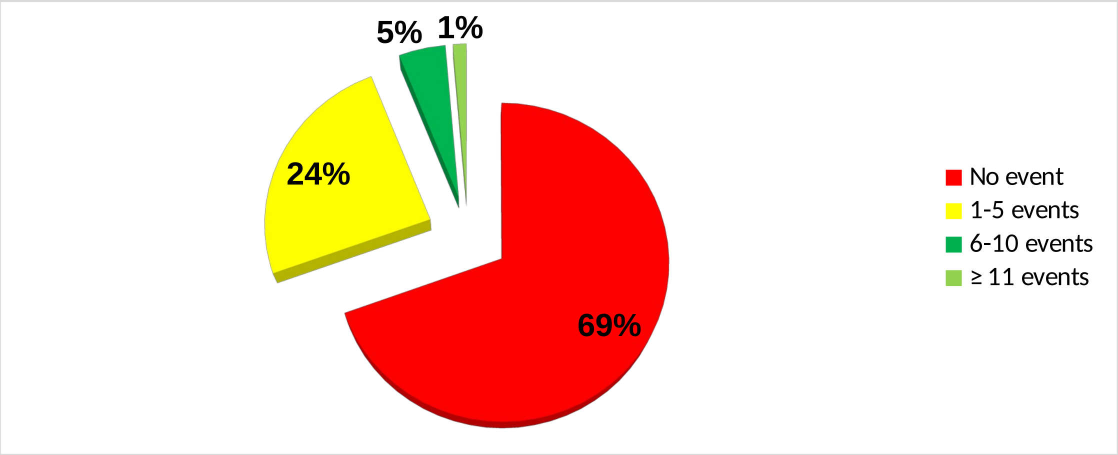
5%
1%
24%
69%
No event
1-5 events
6-10 events
≥ 11 events
